# Aging, Gut Microbiota and Metabolic Diseases: Management through Physical Exercise and Nutritional Interventions

**DOI:** 10.3390/nu13010016

**Published:** 2020-12-23

**Authors:** María Juárez-Fernández, David Porras, María Victoria García-Mediavilla, Sara Román-Sagüillo, Javier González-Gallego, Esther Nistal, Sonia Sánchez-Campos

**Affiliations:** 1 Institute of Biomedicine (IBIOMED), University of León, 24071 León, Spain; mjuarf@unileon.es (M.J.-F.); dpors@unileon.es (D.P.); mvgarm@unileon.es (M.V.G.-M.); sroms@unileon.es (S.R.-S.); jgonga@unileon.es (J.G.-G.); menisg@unileon.es (E.N.); 2 Centro de Investigación Biomédica en Red de Enfermedades Hepáticas y Digestivas (CIBERehd), 28029 Madrid, Spain

**Keywords:** dietary intervention, elderly, gut microbiota, metabolic diseases, physical exercise, prebiotics, probiotics

## Abstract

Gut microbiota (GM) is involved in the maintenance of physiological homeostasis, thus the alteration of its composition and functionality has been associated with many pathologies such as metabolic diseases, and could also be linked with the progressive degenerative process in aging. Nowadays, life expectancy is continuously rising, so the number of elder people and the consequent related pathologies demand new strategies to achieve healthy aging. Besides, actual lifestyle patterns make metabolic diseases a global epidemic with increasing trends, responsible for a large mortality and morbidity in adulthood and also compromising the health status of later stages of life. Metabolic diseases and aging share a profile of low-grade inflammation and innate immunity activation, which may have disturbances of GM composition as the leading mechanism. Thus, GM emerges as a therapeutic target with a double impact in the elderly, counteracting both aging itself and the frequent metabolic diseases in this population. This review summarizes the role and compositional changes of the GM in aging and its modulation through nutritional interventions and physical exercise as a strategy to counteract the aging process and the related metabolic diseases.

## 1. Introduction

Life expectancy has been on the rise constantly since the second part of the last century and it is projected to continue this trend in the next years [1,2]. In developed countries, men can easily reach the age of 79 and this number is slightly higher for women (up to 84 years old) [1], thus adults over 65 years old would constitute 20–25% of total population in Europe and the US by 2030 [3]. Modern societies need to deal with an increasingly aging population, being a major burden for public health systems due to the huge number of associated comorbidities associated with older adults. This is worsened by the so-called drugs-related problems, as polypharmacy is common thorough elder people, demanding even more medical resources and reducing the efficacy of prescribed treatments [4]. Thus, exploring methods that can guide us towards a healthy aging besides neglecting pharmacological and surgical therapies is a promising research strategy.

The term “age-related diseases” (ARDs) [5] has been proposed to describe a spectrum of chronic disorders that usually manifests in the elderly, despite the fact that its triggering may be accelerated by circumstances occurring in the adult age or even in childhood, frequently associated to bad nutritional patterns and a sedentary lifestyle. Therefore, although a good management of ARDs may need prevention strategies before the onset of these pathologies, targeting the mechanisms that contribute to aggravate them should also be considered [6,7]. An active lifestyle including habitual physical exercise is widely accepted as a valid method to promote health in all stages of life. The benefits of exercise include body weight reduction, counteraction of blood hypertension and dyslipidemia or insulin resistance attenuation, all of them factors contributing to prevent cardiovascular events and metabolic disorders [8]. Moreover, increased physical activity has a positive effect on mental health and cognitive function [9,10]. These benefits could be especially important in the elderly as this population have the higher sedentary rates, spending the vast majority of their daily time sitting [11].

A wide amount of research has focused on gut microbiota composition and functionality and its implications for the maintenance of a healthy status. The gut microbiota (GM) isdefined as a metabolic ecosystem of a huge variable microorganisms which habit in the gastrointestinal tract and establish a symbiotic relationship with the host. These microorganisms are, principally, bacteria, but it is also important the role and the presence of virus, fungi or archaeas. The abundance of the microbiota in humans is ten times higher than their somatic and germ line cells [12], reaching concentrations of 10^11^ colony-forming units (CFU) per gram of gut content in the colon [13]. In fact, the genes of this microbiota, called “microbiome”, are 150 times larger than the human genome [13].

Colonization of the intestine begins even before birth and it is susceptible to high variability due to its plasticity in the early stage of life, remaining more stable in the rest of the lifetime. The changes which take place in adulthood reflect environmental factors such as diet, antibiotic consumption, etc. Given its crucial role to maintain body homeostasis, disturbances of the GM are associated with a growing group of pathological conditions, being a potential initiating mechanism rather than a simple symptom. In this context, microbiota-based therapies are being considered as a potential strategy or at least an adjuvant for conditions like obesity, in the form of prebiotic, probiotic or symbiotic administration. Furthermore, the impact of lifestyle on the gut microbiota reinforces the therapeutic role of nutrition and physical activity, not only by their side effects due to body weight loss but to a direct action reshaping the microbiota.

In this review we aim to deep in the role of gut microbiota as a target for therapeutic strategies based on physical exercise and nutritional interventions in the elderly, with a special focus on the management of obesity and related metabolic diseases.

## 2. Metabolic Diseases in the Elderly

Obesity and associated comorbidities, including metabolic syndrome, non-alcoholic fatty liver disease (NAFLD) or type 2 diabetes mellitus (T2D) have experienced a dramatic increase over the last few years. Despite regional disparities, almost a third of the world’s population are overweight or obese, of which a range between 30–37% present NAFLD [14], increasing to 90% in morbidly obese people [15]. Meanwhile, 61% cases of T2D could be attributed to overweight [16]. Moreover, the risk of non-metabolic diseases, including cardiovascular, musculoskeletal diseases or cancer, also increases with obesity [17]. The prevalence of overweight and obesity tends to augment with age, despite a slight decrease in the population over 65 years [18]. This trend may be influenced by the difficult definition of obesity in the elderly due to the confluence of two naturally occurring processes: a progressive increase in fat content, particularly abdominal fat, and the loss of muscle mass and strength with aging (sarcopenia). Therefore, the traditional definition of obesity based on body mass index (BMI) is less accurate in this group as it tends to underestimate the effects of body composition. This is the reason that the term sarcopenic obesity has arisen to characterize the concurrence of increasing obesity rates in an aging population [19].

Adverse effects of obesity in older adults may be different to those observed in young and middle-aged individuals. Some authors have claimed an “obesity paradox”, as increased mortality associated to obesity is not always observed in elderly obese patients [20]. This could be attributed to the cofounding effect of unintentional weight loss due to other pathological conditions, a reduced risk to develop complications of obesity established in the elderly, or the above mentioned underestimation of body composition changes [3]. In fact, sarcopenic obesity may suppose a synergetic effect between both conditions, maximizing the risk for morbidity, mortality and physical disability [21]. Furthermore, obesity in the elderly could exert even more negative effects, for example being a threat to cognitive functionality. In a study on 1100 Chinese subjects aged 60 or above, abdominal obesity was associated to a higher risk of cognitive impairment independently of other environmental factors or metabolic conditions [22].

The role of inflammation on aging and its relationship with metabolic diseases is noteworthy. Both aging and metabolic diseases are accompanied by an inflammatory environment (metaflammation and inflammaging, respectively), thus potentially contributing to each other in a vicious cycle [23]. This shared low-grade inflammatory state between aging and metabolic diseases could be mediated by the innate immune system. In fact, mediators of the innate response-like pattern recognition receptors (PRRs) may act as sensors of a large number of molecules (pathogen-associated molecular patterns (PAMPs), and/or damage-associated molecular patterns (DAMPs)), including saturated fatty acids from the diet which contribute to obesity and T2D development, or cell debris and misfolded or oxidized proteins that are potential stimuli in inflammaging [24]. Moreover, microbial-derived products are involved in both processes, being potent ligands for PRRs. This, together with the plasticity of the gut ecosystem thorough lifetime points to the GM as the possible link between metabolism and aging [23]. The gut-liver axis may exert a critical role in this puzzle, as disturbances of the intestinal barrier are strongly associated to metabolic diseases [25]; besides, intestinal permeability tends to increase with age, favouring the leakage of potential immunomodulatory substances to the systemic blood and the liver [26]. These evidences reinforce the importance of targeting gut microbiota as a therapeutic strategy for metabolic disease, also in the elderly.

## 3. The Gut Microbiota as a Key Factor in the Development of Metabolic Diseases

In recent years, gut microbiota has been identified as a contributor factor in the development of many pathologies such as immune disorders (i.e., psoriasis or asthma), allergies, cardiovascular and neurodegenerative diseases, obesity, NAFLD, metabolic syndrome, T2DM or even some cancers [27]. Thus, microbiota-related research has risen drastically to understand the microbiota-host interaction and to know the exact role of the gut microbiome in these pathologies, with the purpose to identify new therapeutic strategies.

Microbiota diversity and composition alteration have been associated to obesity development, the manipulation of them being feasibly useful on therapy [28]. Host energy harvest and host energy metabolism are modulated and regulated by gut microbiota and its metabolites. In fact, it has been observed that gut microbiota is capable of promoting the intestinal absorption of monosaccharides, increasing as a consequence of *de novo* hepatic lipogenesis and triglycerides accumulation in the adipocytes [29]. Bäckhed et al. [30] conducted the first study that demonstrated the role of gut microbiota in the progression of obesity. They observed that conventional mice had 42% more total body fat compared with germ-free (GF) mice, instead of second ones having a higher caloric consumption. Therefore, when GF mice were colonized with normal microbiota from conventional animals, they suffered insulin resistance and an increase of 60% in their body weight despite a reduction in their food intake [31]. Moreover, human fecal microbiota transplantation to GF mice demonstrated that obesity-related microbiota phenotype could be transferred, confirming definitively that gut microbiota is a key factor in body weight management and fat deposition [32]. Nevertheless, these results could not be reproduced for all diets and mouse strains; more studies are necessary to understand how the gut microbiota participates in obesity [33]. It has been observed that microbiota composition of obese children is different in comparison with lean children [34,35] and it has been suggested that microbiota profile during early stages of life is hugely important to becoming obese in adult stage [36]. A great effort has been made to establish a microbial signature of obesity looking for a potential causative role of a determined bacterial taxa. The first evidence pointed to a higher abundance of bacteria of the phylum *Firmicutes* and a lower abundance of *Bacteroidetes* as a hallmark of obesity both in humans and rodents [37,38], although later research sometimes described the opposite, still being a controverted issue. Several reports of a reduction of bacterial diversity on obese subjects have also been released [39,40]. Related to specific modifications of microbial taxa at a deeper level than phylum, an increased abundance of *Lactobacillus* [41] or LPS producing bacteria belonging to the phylum *Proteobacteria* [42,43] and the depletion of potential beneficial bacteria like several species of *Bifidobacterium* genus, *Faecalibacterium prausnitzii* or *Akkermansia muciniphila*, has been described [44]. To sum up, a recent systematic review confirmed the widely assumed increased *Firmicutes* to *Bacteroidetes* ratio in obese humans, with a vast majority of studies reporting similar results; however, a great variability was observed regarding the other findings [45].

On the other side, the implication of gut microbiota in many liver diseases, such as non-alcoholic and alcoholic fatty liver, cirrhosis, hepatic encephalopathy or even hepatocarcinoma (HCC), has been widely studied [46,47,48]. Le Roy et al. [49] suggested for the first time the role of gut microbiota in NAFLD progression, due to the steatosis development in GF mice as consequence of fecal microbiota transplant from high-fat diet (HFD)-fed mice. Moreover, microbial products of fermentation such as ethanol have been pointed to induce obesity and facilitate the development of fatty liver disease, as well as gut microbiota could promote hepatic steatosis through the modulation of bile acid metabolism [36]. The main hypothesis to explain how gut microbiota alteration, known as intestinal dysbiosis, affects to NAFLD development is based on the fact that the major part of liver’s blood is supplied by the portal vein, which connects the gut with the liver in the known “gut-liver axis” and allows one to enter endotoxins and microbial-products to the liver. On a healthy status, these bacteria metabolites and toxins are counteracted by an immune system. However, high-fat diets, sedentary habits or intestinal dysbiosis are capable of disrupt intestinal barrier and, as consequence, alter gut permeability, allowing the entrance of high amounts of bacteria and their products to the liver [33] and increasing metabolic endotoxemia. One of the most important bacteria endotoxins is the lipopolysaccharide (LPS), which activates the nuclear factor kappa B (NF-κB) signaling pathway through Toll-like receptor 4 (TLR-4) and induces the consequent liberation of pro-inflammatory cytokines such as tumor necrosis factor alpha (TNF-α) or interleukin 6 (IL-6). All these microbiota alteration-originated changes lead to the promotion of an inflammatory hepatic state that facilitates and aggravates NAFLD development [33,50]. To establish a microbiota profile on NAFLD patients is difficult due to the spectrum of liver lesions beheld under this term and the confounding effect of the comorbidities associated to NAFLD. Some of the microbial changes observed in NAFLD were shared with obesity, as NAFLD is seen as another piece of the so-called metabolic syndrome. Thus, increased *Proteobacteria* phylum abundance or reduction in beneficial *Faecalibacterium* genus has been reported in human NAFLD [51]. Moreover, microbiota composition in NAFLD may evolve, along with the worsening of the condition. Comparing patients from different stages of liver damage suggests that *Bacteroides* may predominate in the microbiota of patients with NASH, while *Ruminoccocus* would be a signature of advanced liver fibrosis and cirrhosis [47]. Increased abundance of these taxa may also be associated with the development of cirrhosis-related HCC, along with the depletion of *Akkermansia* and *Bifidobacterium* [46].

The participation of the gut microbiota in T2D has been evidenced too. Comparisons of microbiota profiles of human adults with and without T2D demonstrated the existence of a completely different gut microbiota pattern in this disease, which is characterized by an increase of *Bacteroidetes* and *Betaproteobacteria* and a reduction of *Clostridia*. Moreover, a tendency to decrease *Clostridia* levels with the increasing plasma glucose levels in T2D disease was also observed [52]. Qin et al. [53] developed a metagenome-wide association study (MGWAS) to identify metagenomic markers in T2D and they concluded that patients with T2D had a moderate gut dysbiosis characterized by an increase in opportunistic pathogens and a reduction in butyrate-producers bacteria. Thus, reshaping the gut microbiota may be an alternative in the management of T2D. Tonucci et al. [54] concluded in their double-blind, randomized and placebo-controlled trial that the administration of probiotics could be a new approach for the prevention and treatment of T2D development because of its capacity to improve the glycemic control. Moreover, one of the main pharmacological strategies to deal with T2D, metformin, is capable of modify the gut microbiota composition, as Wu et al. [55] demonstrated in their research. They also showed that the fecal microbiota transplantation from metformin-treated patients to germ-free mice improved the glucose tolerance of the animals, suggesting that this altered microbiota could be taking part in the metformin effect.

This evidence reinforces the role of gut microbiota in metabolic diseases, which are constantly increasing worldwide. Due to the importance of healthy aging, the study of gut microbiota composition in the elderly and its modification by feasible therapies to prevent or treat metabolic diseases remains essential.

## 4. Gut Microbiota in the Elderly

As previously stated, even in physiological conditions, the gut microbiota is a dynamic ecosystem. The composition of the gut microbiota is based on permanent and transitory bacterial species of 17 different phyla such as *Firmicutes*, *Bacteroidetes* and *Proteobacteria*, which can reach as much as 70%, 30% and 5% of the total abundance, respectively, between others [56]. This composition changes depending on the anatomic region of the gastrointestinal tract, due to pH, secretions, motility or substrate availability. A gradual increase of bacterial concentration and complexity exists through the stomach and the gut, reaching the maximum in the colon. Moreover, GM experiments with taxonomical and functional changes during the life of an individual since the prenatal period. The microbiota colonization on the gastrointestinal tract may be started in utero with the placenta and amniotic fluid microbial communities of the mother, as recent research has observed when comparing these microbial populations with the meconium ones [57]. Microbiota profile constitution is affected by numerous factors, such as genetic components, type of delivery (vaginal or cesarean), the feeding (breast-feeding or formula-feeding) or antibiotics and/or probiotics consumption during the first days of life. The initial gut microbiota of infants is a quite instable simple structure dominated by bifidobacteria [58] and is in continuous change until the age of three years. At this moment, the microbiota profile is established and acquires an adult pattern that is relatively stable over time. Nevertheless, there are many causes that can modify this adult profile, for instance lifestyle, exercise, dietary patterns, stress or pathophysiology. Microbiota changes drastically in the elderly and these age-related changes are directly correlated with an inflammatory pattern linked with many diseases. Generally, these changes are orientated to a loss of diversity, a reduction of the abundance of beneficial bacteria such as those which produce short-chain fatty acids (SCFAs) [59], a change in the dominant species or an increase of enteropathogens [60]. All of these modifications are associated with physiological changes in the gastrointestinal tract and in dietary patterns, and with a decrease in the immune system function [61], an increase in the inflammatory state and a feasible contribution to the progression of diseases [59] and frailty [61]. Regarding the changes in dietary choices in the elderly, reductions in taste, dentition, chewing ability and intestinal transit time are factors that contribute considerably [62].

Some researchers have focused on establishing the specific age-related microbiota profile. At phylum level, *Firmicutes* is predominant in adults, being reduced in the elderly, whereas there is some discrepancy about the increase or decrease of *Bacteroidetes* phylum with age [61,63,64]. Moreover, high levels of *Proteobacteria* phylum especially *Enterobacteriaceae* family [63] and *Clostridia* class [64]), as well as a decrease of *Actinobacteria* (especially *Bifidobacterium*, a genus with intestinal protective capacity) have been reported in old people [61,63,64,65]. In fact, inflammatory markers such as IL-6 or IL-8 have been associated with an enrichment in *Proteobacteria* phylum, which increases with age [66]. Biagi et al. [66] identified in an elder population in Italy a decrease in bacterial diversity, as well as a change in the relative proportion of *Firmicutes* phylum, an increase of *Bacili* and low levels of *Clostridium* cluster XIVa. This reduction in the abundance of *Clostridium* cluster XIVa was corroborated by later studies [58]. Moreover, Rahayu et al. [64] analyzed the gut microbiota composition of 80 volunteers from Bali and Java arranged in two groups depending on the age (25–45 age old and 70 years old), identifying a reduction of *Bifidobacterium, Prevotella* and *Lactobacillus plantarum* taxa and an increase in *Enterobacteriaceae* and *Lactobacillus reuteri* in the elderly subjects). Furthermore, Bian et al. [67] developed a study with 1095 healthy volunteers from different cities of China and showed a decrease of *Bifidobacterium* and *Bacteroides* genera in older subjects, whereas *Dorea, Clostridium* and *Marvinbryantia* genera were increased in that population. In this research, *Faecalibacterium* genus was identified as a core and stable microorganism among life. Additionally, Claesson et al. [68] observed that low levels of diversity were correlated with inflammatory markers, frailty and impaired health parameters, as well as diet patterns. Frailty has also been associated with a low abundance of butyrate-producers like *Faecalibacterium prausnitzii* [63,69], *Lachnospiraceae* family and *Roseburia* genus, whereas there are some species that associate positively with frailty, such as *Eggerthella dolichum* or *E. lenta* [69].

In spite of the great inter-variability among studies, there are some taxa which may be less susceptible to be modified by external factors and may constitute the core of gut microbiota composition in the elderly. The reduction in the abundance of *Ruminococcus*, *Blautia* or *Clostridium* cluster XIVa and *Clostridium* cluster IV and the major prevalence of facultative anaerobes like *Escherichia coli* are changes that have been observed in the elderly population across many studies [70]. Even so, it is difficult to establish a unique aging gut microbiota composition profile, due to many factors that modulate this internal ecosystem such as the ethnicity, lifestyle, dietary patterns, host genetics, the presence of comorbidities or even methodological tools. Those reasons reveal the importance to do more studies to reach a homogeneous aging gut microbiota signature.

In the elderly, the presence of comorbidities is a very common situation that requires polypharmacy in order to improve the health status. Thus, not only the comorbidities but also the polypharmacy are factors that modify drastically the composition and diversity of microbiota. In fact, Ticinesi et al. [71] studied the differences between a cohort of 76 elderly hospitalized and multimorbid patients and a group of 25 healthy active elderly volunteers. The β-diversity index showed that the microbiota profile of hospitalized patients was significantly different in comparison with non-hospitalized group. The number of drugs was negatively correlated with Chao-index α-diversity and with the taxa *Massilia* and *Lachnospiraceae*. Furthermore, *Coprobacter*, *Helicobacter* and *Prevotella* were positively correlated with polypharmacy. The hospitalization is another factor that modifies gut microbiota composition, characterized by a substantial decrease in *Faecalibacterium prausnitzii*, *Desulfovibrio* spp. or *Bifidobacterium*, between others, and a major increase of the abundance of enterobacteria [63]. Moreover, Claesson et al. [68] and Jeffery et al. [62] identified in a study which compared the gut microbiota of Irish elderly by their type of residence (community-dwelling, one day at hospital, short-term rehabilitation and residential care) a relationship between the institutionalization of elderly people and an increase of *Firmicutes* phylum and *Parabacteroides*, *Eubacterium*, *Anaerotruncus* and *Coprobacillus* genus, as well as a reduction in bacterial diversity and the abundance of short-chain fatty acid (SCFAs) producers. These results are in agreement with previous reports, indicating that the microbiota profile related to age is aggravated by polypharmacy, reducing the number of SCFAs producers such as *Lachnospiraceae* family and increasing the abundance of some enteropathogens like *Helicobacter*.

It is important to consider the importance and the difference between biological and chronological age, being first physiological age, which takes into consideration many issues such as lifestyle or environmental and genetic factors, and the second one the number of years a person has been alive. Bacterial diversity has been negatively correlated with biological age, but not with chronological age. Moreover, *Ruminococcus*, *Coprobacillus* and *Eggerthella* genera have been associated positively with biological age, independently of the chronological one [59]. Related to that, many studies have identified the microbiota profile of centenarians—those people close to 100 years old—showing a specific healthy composition closer to adults’ pattern and remarkably different to that commonly observed in elders over 65 years. Wang et al. [72] described the composition of gut microbiota of centenarians in East China and observed that the α-diversity was significantly increased, as well as the genus *Escherichia* and *Roseburia* in centenarians. Moreover, volunteers more than 100 years old also showed a decrease in the abundance of *Lactobacillus*, *Butyricimonas*, *Coprococcus*, *Parabacteroides*, *Akkermansia*, *Sutterella* and *Faecalibacterium* genera compared with the groups between 80 and 99 years old. Afterwards, Wang et al. [73] conducted a similar study in a larger population and described that community richness and α-diversity was significantly lower in the 65–70 years age group compared with the 90–99 and the 100+ year age groups. An increase in the relative abundance of *Synergistetes* phylum (with special mention of *Prevotellaceae*, *Lachnospiraceae* and *Porphyromonadaceae*) was observed in the longevity group compared with the younger elderly group. Similar results related to the increase of microbial diversity and *Porphyromonas* genus in centenarians were observed in a study of 367 Japanese volunteers [65]. Furthermore, Kim et al. [74] identified a minor relative abundance of *Faecalibacterium* and *Prevotella*, as well as an increase of *Escherichia* and *Proteobacteria* in centenarians of South Korea. Additionally, Kong et al. [75], considering the results of their own Chinese cohort and the results previously reported by Biagi et al. [76], identified an enrichment of *Clostridium* cluster XIVa, *Akkermansia*, *Ruminococcaceae* and *Christensenellaceae* in long-living groups. While many genera of *Clostridium* cluster XIVa are producers of SCFAs, *Akkermansia* and *Chistensenellaceae* have been identified as good metabolic health-related bacteria, associated with healthy homeostasis and immunomodulation. This suggests a tendency in the microbiota profile of centenarians towards a healthy and anti-inflammatory status [74,75]. Moreover, related to SCFAs producers, the microbiota profile of centenarians showed an increase in some butyrate producers (*Anaerotruncus colihominis* and *Eubacterium limosum*) and a decrease in others (*Ruminococcus obeum*, *Roseburia intestinalis*, *E. ventriosum*, *E. rectale*, *E. hallii*, *Papillibacter cinnamovorans* and *Faecalibacterium prausnitzii*), suggesting with these differences the presence of bacteria characteristics of longevity [77]. The association of longevity with *Ruminococcus*, a genus known as a SCFAs producer and with an important role in gut protection, is still contradictory [73]. All of these findings related to gut microbiota composition in elderly and in centenarians are summarized in Table 1.

In these terms, not only the composition but also the metabolic pathways of microbiota change with age. Collino et al. [78] identified some alterations in a Northern Italian population linked to age, such as low concentrations of tryptophan and lysophospatidylcholines and increased levels of sphingomyelins and phospatidylcholine 32:0. On the other hand, some plasma metabolomic patterns such as lipids and amino acids have been related to health span markers in elderly [79]. Nevertheless, it has been observed that phosphatidylinositol, glycosphingolipid and N-glycan biosynthesis signaling pathways are increased in centenarians, all of them being associated with anti-inflammation and healthy status of gut microbiota [74]. Low levels of markers of lipid peroxidation, as 9-hydroxy-octadecadienoic acid (9-HODE) and 9-oxo-octadecadienoic acid (9-oxoODE), have been identified in longevity phenotype in a population of Italy [78], while centenarians in China showed high levels of SCFAs and total bile acids [80]. These results seem to reinforce the existence of a specific altered microbiota pattern in th+++e elderly with the particularity of a healthy microbiota composition and functionality in centenarians, with more research being necessary to elucidate such patterns.

## 5. Gut Microbiota Targeted Interventions in the Elderly

The field of gut microbiota manipulation, although relatively new, has attracted a lot of attention and the literature about this issue is constantly growing, however research focused on elderly population is still scarce. Dietary interventions and bioactive supplements have been explored as potential therapies for obesity and related diseases via gut microbiota composition alterations, obtaining promising results. More recently, physical exercise has demonstrated the ability to reshape gut microbiota, being another piece behind beneficial effects of this approach in the treatment of metabolic diseases [81]. Given the role of the microbiota in the elderly, it would be useful to elucidate the capacity of such alternative therapeutics to counteract both metabolic and aging-associated alterations. Here, we summarize studies assessing gut microbiota targeted interventions in the aged population (Table 2) or in animal models of aging, with or without the presence of obesity and associated comorbidities.

### 5.1. Dietary Intervention

Diet is the principal contributor to determine the composition of the gut microbiota, although the great interindividual variability in older adults overwhelms the effects of other possible covariables [62,68]. This point is especially important due to the limitations in the diet of elder people which may suffer from low diversity and insufficient nutrient intake, since some type of foods are usually excluded due to their low palatability or difficulty to chew [93]. Thus, strategies directly guided to modify dietary patterns are more likely expected to be successful to counteract dysbiotic microbiota associated with metabolic diseases. Moreover, the administration of functional foods with prebiotic properties or even live bacteria as probiotics needs to be considered.

Mediterranean diet (MD) is highly appreciated due to its multiple health benefits associated with a lower risk of cardiometabolic diseases occurrence and to a reduced frailty [94]. The microbiota modulatory action of MD in the elderly population has been addressed both in the short term and in a one-year follow-up intervention. Fifteen days of MD in obese women (≥65 years) resulted in an approximately 3% weight loss, accompanied by a shift in microbiota composition towards reduced abundance of *Collinsella* and an increase in that of potentially beneficial bacteria like *Parabacteroides*, *Bacteroides*, *Christensenellaceae* or *Methanobrebrevibacter* [82]. Although nutritional intervention was the main objective of this study, it was a branch of a multi-intervention program that also included physical activity, so despite the short period, synergetic effect of both diet and exercise needs to be considered regarding changes in microbiota composition. Ghosh et al. [83] recently reported the results of a one-year multi-centre study involving elderly subjects under dietary guidance based on MD. Following random forest models, they identified a subset of taxa correlated with the adherence to the diet which respondedeither in a negative or positive trend. The taxa positively modified by the diet comprised well-known beneficial bacteria including *Faecalibacteriumn prausnitzii* and *Roseburia*, several species of *Eubacterium* genus, *Bacteroides thetaiotaomicron*, *Prevotella copri* and *Anaerostipes hadrus*. On the contrary, diet negatively regulated OTUs belonging to *Ruminococcus torques*, *Collinsella aerofaciens*, *Coprococcus comes*, *Dorea formicigenerans*, *Clostridium ramosum*, *Veillonella dispar*, *Flavonifractor plautii* and *Actinomyces lingnae*. In summary, MD seems to be associated in aged people with the increase in beneficial bacteria, some of them SCFA’s producers (*Faecalibacteriumn prausnitzii*, *Roseburia*), and the reduction of potential pathobionts (*Collinsella*).

Negative results of a dietary intervention in an elderly population in terms of gut microbiota composition modification were reported by Mitchell et al. [84]. Based on the recommendations of increasing protein intake in the elderly [95], 30 individuals aged 70 or older were allocated either to a group consuming the recommended dietary allowance (RDA) of protein or to a group consuming twice the RDA for 10 weeks. No differences in the microbiota composition or in the faecal volatile organic compounds were detected as a consequence of the intervention. Thus, a possible resilience of gut microbiota on the elderly to be modified by the diet should be considered, emphasising the usefulness of supplements which may increase the effectiveness of dietary interventions.

### 5.2. Prebiotic and Probiotic Supplementation

Prebiotic and probiotic supplementation may also be used to reverse aging-induced changes in gut microbiota. Human studies over the period 2005–2017 involving prebiotics, probiotics and their combination in healthy aged populations were collected and summarize elsewhere [61]. Briefly, interventions with *Bifidobacterium* species alone or in combination with prebiotics were the majority, but limited effects on gut microbiota composition were detected besides the increase in the relative abundance of *Bifidobacterium* genus and the potential reduction of opportunistic entheropathogens [96,97,98,99]. A recent study with prebiotics considered the possible implication of institutionalization in the elderly. The research, conducted over 3 populations (healthy young adults, community dwelling elderly and institutionalized elderly), reported that the microbiota from institutionalized elderly adults seemed to be more responsive to changes due to a prebiotic mix administration for 26 weeks [85]. *Parabacteroides*, *Clostridium* cluster IV, *Ruminoccocaceae* family and *Phascolarctobacterium* increased after prebiotic administration in this group in the short term (week 13). Thus, the efficacy of prebiotics to modify gut microbiota composition in the elderly could be related to frailty, but their effects on inflammation were very modest despite the long intervention period. Regarding probiotics, a mix of two *Bifidobacterium* species in an over 65 years old South Korean population, achieved a gradual and significant reduction of *Allisonela*, *Eubacterium*, *Clostridiales* order and *Prevotellaceae* family after 12 weeks [86]. The reduction of *Prevotellaceae* abundance is in contrast with the dynamics of this taxa under other treatments as exercise or MD where this genus shows the opposite trend. Nevertheless, probiotics were associated to an improvement in cognitive function and reduced stress, which strengthens the importance of the gut-brain axis in the elderly.

The previously mentioned study by Cancello et al. [82] also analyzes the impact of the widely used probiotic mix #VSL3 on obese women in combination with the MD intervention over 15 days following the initial phase of dietary intervention alone. Besides, significant increases in the bacterial genus present in the cocktail, the promising probiotic *Akkermansia* was found to increase after this period. No further research on the effect of probiotics in the microbiota of elderly obese humans has been carried out to date, however in an aged mouse model of HFD-induced obesity, the administration of a probiotic cocktail (5 *Lactobacillus* and 5 *Enterococcus* strains) was able to counteract metabolic syndrome and deeply reshape the gut microbiota [100]. Both α and β diversity were modified in response to the probiotic mix as well as phylum like *Firmicutes* and *Verrucomicrobia* which were increased and decreased, respectively. Going down to the species level, the abundance of *Akkermansia muciniphila*, *Peptococcus niger* and *Ruminicoccus gnavus* decreased, while two species of *Clostridium*, *Roseburia faecis*, *Enterococcus lactis*, *Bacteroides salanitronis*, and *Lactobacillus rhamnosus* increased. The decrease in *A. muciniphila*, bacteria known for its health promoting effect, is noteworthy. This study also provides an extent demonstration of gut-liver axis modulation in the beneficial effect of the probiotic, linked to leaky gut prevention and an anti-inflammatory effect, which is valuable both in the treatment of obesity and as antiaging therapy. Furthermore, probiotic treatment was associated to an improvement in physical aptitude of old mice. However, the absence of a control diet-fed group limited the utility of these results as it is not possible to determine which of the negative features and changes in microbial taxa observed in HFD-fed old mice ameliorated by the probiotic are a consequence of the diet or aging.

### 5.3. Physical Activity

Related to the role of exercise in the aged microbiota, some evidences came from studies that assess the relationship between the microbiota and the amount of physical activity routinely performed, or the physical fitness demonstrated in different standardized tests.

In a cohort of volunteers ranging from 65 to 92 years old that met the criteria of functional independence and absence of diseases that severely diminished quality of life, the monitoring of physical activity by electronic devices over a month was employed to identify changes associated to different degrees of activity in several selected bacterial taxa by qRT-PCR [87]. No significant differences were detected in the total counts of any of the bacterial taxa analyzed between more active (energy expenditure >3 metabolic equivalents (METs) for >15 min/day) and less active (energy expenditure >3 METs for <15 min/day) subjects, and only *Bacillaceae* and *Fusobacteriaceae* families differed significantly (increased and decreased, respectively in more active vs. less active) when the results were expressed as relative abundance of the total families detected by massive sequencing. Thus, in this study, despite being associated with improved intestinal health in older adults, routine physical activity seems to have a minor impact on gut microbiota composition. It is worth mentioning that sequencing data provided only went down to the family level, as authors assessed some specific genera and species by PCR techniques; so, possible effects of physical activity at those levels may be underestimated.

Following the other approach, Castro-Mejía et al. [88] classified individuals into two groups (high physical fitness and low physical fitness) according to their performance in the 30 s Chairstand test, BMI and percentage of leg-soft-tissue fat. Multivariant analysis identified a group of variables which can accurately discriminate between groups (dietary sources, plasma and fecal metabolites, microbial taxa, biochemical parameters and steps per day), including higher abundance of *Bifidobacterium adolescentis* and several species of *Chritensenella* genus in high fitness group, whereas the low fitness group microbiota was enriched in *Enterobacterales*. These results point to a complex relationship between the gut microbiota, the dietary and daily activity patterns and the resultant physical fitness. However, the design of the study limited the scope of the observations made. The parameters selected to stratify the participants may have a profound impact on the conclusions as BMI is taken into account to allocate individuals on the low or high fitness group; overweight or obesity are conditions that may underly the differences between groups, overestimating the contribution of physical activity or dietary patterns. Furthermore, the usage of a multivariant analysis approach may not be the most accurate to determine individual effects of the variables analyzed (i.e., gut bacteria) and may be affected by a large number of confounding factors.

There are scarce studies that carry out a controlled training program and report metagenomic analysis results in elderly population. A short-term endurance exercise program in a group of healthy Japanese aged 62–76 years only achieved modest effects on gut microbiota composition. Overall, 5 weeks of 3 sessions/week exercise on a cyclo-ergometer did not modify α or β diversity of bacterial communities. Just *Oscillospira* at the genus level and *C. difficile* at the species level showed an increase and a decrease, respectively, associated to exercise practice; however, the former only was detected in control period of the first-group and did not reach significance after adjusting for confounding dietary factors. Exercise was also associated to a functional modulation of the microbiota with increasing capacity for “genetic information processing” and “nucleotide metabolism”, according to PICRUSt predicted metagenomes [89]. This limited effect of exercise may be attributed to the short training protocol, as significant microbial adaptations to exercise probably require extended periods of time to take place.

Morita et al. [90] carried out a 12-week intervention in healthy elderly women, addressing the effects associated to different approaches to exercise practice. Women allocated to perform brisk walking as a type of aerobic exercise training showed an increase in *Bacteroides* and a decrease of *Clostridium* subcluster XIV at the end of the study, while the control group who performed trunk muscle training alone revealed only an increase of *Clostridium* cluster IX. Thus, opposite to results by *Taniguchi* et al. [89], aerobic exercise training seems to promote a significant change of bacterial composition towards increased *Bacteroides* abundance in an elderly population, correlating with an improvement of cardiorespiratory fitness. Besides the different protocol and duration of the intervention, gender could be another cause of discrepancies as these studies either enrolled only males or only females.

Two studies have addressed the effect of physical activity on an elderly population manifesting components of the metabolic syndrome, either hypertension or overweight. Hypertension is a condition highly associated to obesity and linked to increased risk of cardiovascular events. Patients with hypertension aged 65–80 showed a particular microbiota fingerprint according to its exercise capacity based on its peak oxygen uptake [91]. On one hand, the microbiota of patients with the normal exercise capacity was enriched in members of the class *Betaproteobacteria*, as well as the family *Ruminococcaceae* and the genus *Faecalibacterium*; the latter well known for its potential beneficial effects in the gastrointestinal tract and previously linked to exercise performance [101]. On the other hand, low exercise capacity patients revealed a higher relative abundance of the pathobiont *Escherichia_Shigella* genus, mainly attributed to *Escherichia coli*, and other bacterial taxa like *Lactobacillales* order, *Lachnospiraceae* family and the genus *Blautia*. Moreover, *Lactobacillales* and *Blautia* positively correlated with C reactive protein levels while the opposite was true for *Alcaligenaceae* genus, belonging to *Betaproteobacteria* class; this suggests a relationship between the abundance of those taxa in the low-grade inflammation of elderly and the capacity to perform physical activity. Furthermore, Zhu et al. [92], besides considering differences associated to aging in a large population from 18 to over 70 years old, analyzed the effect of exercise in the older groups and additionally compared overweight elderly individuals that performed regular exercise with those who never or rarely were involved in physical activity. The results obtained revealed that increasing amount of exercise gradually reshapes gut microbiota in over 70-year-old individuals towards that of adults (18–60 years), dose-dependently modifying the relative abundance of 13 families and 3 phyla, including the increase in the potentially beneficial *Actinobacteria* phylum and *Bacteroidaceae* family. The comparison within the overweight elderly population identified a reduced α-diversity at the OTU level associated to regular exercise practice. At the phylum level, exercise tended to revert the changes associated to overweight phenotype showing higher abundance of *Bacteroidetes* and *Tenericutes* and reduced *Verrucomicrobia*, *Cyanobacteria* and *Firmicutes*, although no significance was reached. Moreover, relative abundance of *Turicibacteraceae* family significantly increased in the regular exercise group, while that of *Pseudomonadaceae*, *Oxalobacteraceae*, *Odoribacteraceae*, and *Barnesiellaceae* showed and opposite pattern. This is the first study reporting differences associated to exercise signature in an elderly overweight population; however, the experimental design and the different comparisons presented make it difficult to follow and interpret the results despite the big sample size. Although limited differences were observed, the results suggest that daily or regular exercise may help to counteract the obesity associated dysbiosis in this population, including remarkable findings like a trend towards a lower *Firmicutes* to *Bacteroidetes* ratio. Overall, to date, the impact of physical activity on gut microbiota composition in an elderly population has been poorly investigated. The studies carried out describe really modest differences between more and less physically active older adults and the same was reported in the two unique studies involving controlled exercise programs. Moreover, the conflicting issue of BMI in the elderly population may mask the results obtained.

Some insights could also be extracted from studies on aged animals or models of accelerated aging related to physical activity impact on gut microbiota composition. It was demonstrated that aerobic capacity could be transmitted by selectively crossbred rats with either low or high aerobic capacity (LC and HC, respectively), resulting in a different susceptibility to diet-induced obesity, adiposity deposition and insulin resistance. Moreover, this phenotype may be driven by differences in gut microbiota composition [102]. Recently, the impact of aging in this model has been assessed, revealing age-dependent differences between HC and LC. Old adult HC rats were characterized by a decrease of *Bacteroidetes*, *Spirochaetes* and *Deferribacteres* at the phylum level, an increased abundance of the genera *Prevotella*, *Mucispirillum* and *Treponema*, while that of *Phascolarctobacterium* and unknown genera of *Erysipelotrichaceae* were reduced respect its age-matched LC counterparts [103]. It is remarkable that old HC rats had a significant lower body weight that could be either cause or consequence of this different microbial signature. On the contrary, no differences in several inflammation and innate immunity mediators were found between both aged groups. The findings of this study could help to understand how exercise capacity may influence age-associated changes in IM composition. Further research is needed to understand this relationship in the elderly, as the animals employed could only be considered adults in terms of human age (10 months is roughly equivalent to 25–30 human years). Houghton et al. [104] assayed the effect of a long term exercise protocol (up to 11 months) in mice with mitochondrial dysfunction (*PolgA^mut/mut^*) as a model of accelerate aging. Mitochondrial dysfunction was associated to changes in gut microbiota at the phylum level with reduced *Bacteroidetes*, while *Firmicutes* and *Proteobacteria* increased, although these differences tended to be attenuated over time. Moreover, the effect of natural aging in both control and mutant mice revealed a reduction of α-diversity, in terms of total different OTUs, in *PolgA^mut/mut^* mice and some changes at genus level, including a higher *Alistipes* and lower *Lactobacillus* relative abundance were maintained over time. Related to the effect of exercise in these mice, there were controversial results as exercise tended to ameliorate reduction of α-diversity, increased *Bacteroides* and reduced potential harmful bacteria like *Helicobacter*, however, *Mucispirillum* and *Desulfovibrio*, both genera associated to inflammation and gastrointestinal disorders, were significantly increase in the exercised group. Although this finding could draw attention over a potential negative effect of exercise on intestinal health in the elderly, this could be a feature of mitochondrial dysfunction induced by the model employed, whose influence may be smaller in humans.

Germ-free mice have proved their usefulness in the microbiota research and have been also employed to identify bacterial taxa associated to a high degree of physical functionality on sedentary older adults (70–85 years) [105]. Microbiota of the participants with the better functionality measures consistently showed an increased abundance of *Prevotellaceae* family and the genus *Prevotella* and *Barnesiella*, whereas a decrease in *S24_7* family was detected across two repeated analysis within a month. Other significant differences were only reported in a single time point. Interestingly, the bacterial taxa enriched in the high functionality group replicated the same pattern in germ-free recipients of six selected donors for each group, resulting in a significantly higher grip strength in mice colonized with stools from high functionality donors, which suggests that these taxa may participate somehow in muscle maintenance. It is interesting the role of *Prevotella* against frailty and its relation with physical activity, as this genus has been found to be increased in the previously mentioned study by Pekkala et al. [103] in high fitness capacity rats and in human athletes or forced exercised rats [106,107].

Physical activity is undoubtedly associated to a healthy aging and to the counteraction of metabolic disorders. However, the modulation of gut microbiota composition as a direct mechanism involved in its health benefits has been barely investigated in both conditions, and more studies are needed to elucidate its role in obese older humans.

## 6. Conclusions

**Figure 1:**
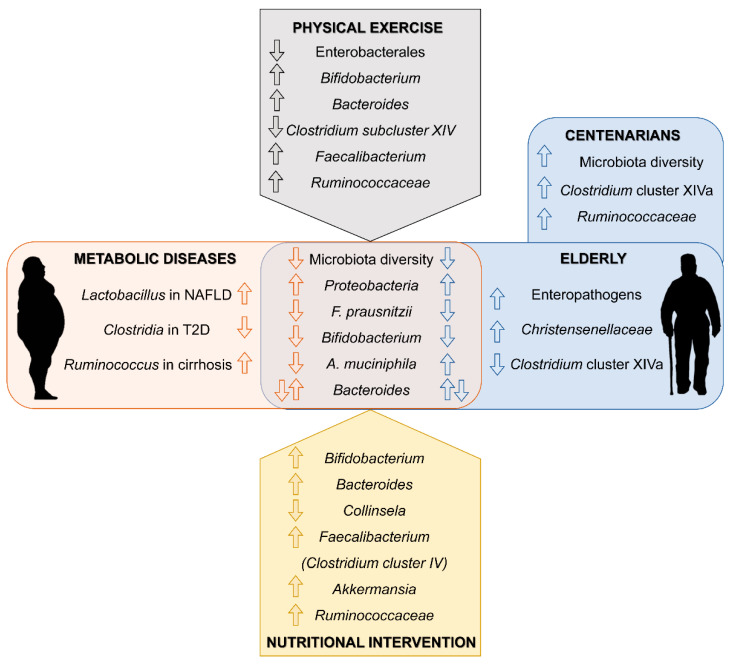
Main findings in gut microbiota composition linked to metabolic disease and aging (center). Summary of related changes observed in response to physical activity and nutritional interventions in elderly individuals (top and bottom respectively).

Gut microbiota is implicated in a diversity of physiological and pathological processes. Its role in obesity and related metabolic diseases is well recognized and pointed out as a promising therapeutic target. Moreover, the microbiota of elder population shows a particular microbial signature that links the natural process of aging to alterations of gut microbiota composition. Thus, in the elderly, strategies to modulate gut microbiota could have a double target: to counteract intestinal dysbiosis induced by metabolic diseases and to reshape the microbial communities associated to aging towards a healthy young-type microbiota (Figure 1). Probiotics and prebiotics are the main direct therapy targeting gut microbiota. However, lifestyle guidelines could indirectly be beneficial through its modulatory effect on gut microbiota. More human studies are needed in aged and metabolically unhealthy population to design microbiota-based therapeutic approaches that take advantage of the relationship between gut microbiota, metabolic diseases and aging.

## Figures and Tables

**Table 1:** Gut microbiota composition of the elderly (≥60 years old) and centenarians (≥99 years old).

Reference	Subjects	Methodological Approach	Main Findings in Gut Microbiota Composition	
[67] Biagi et al. 2010	84 subjects from Northern Italy (50F, 34M). Young adults (20–40 years old) **(Y)**, elderly (60–80 years old) **(E)**, centenarians group (99–104 years old) **(C)** and offspring of the centenarians (59–78 years old) **(F)**	HITChip analysis and qPCR (16S rRNA)	**Elderly**	**Centenarians**
			↑ *Akkermansia muciniphila*	↓ α-diversity index ↑ facultative anaerobes from *Proteobacteria* phylum (*E. coli, Haemophilus, K. pneumoniae* or *Pseudomonas*) and *Bacilli* class (*Bacillus* or *Staphylococcus*) ↓ *Clostridium* cluster XIVa ↓ bifidobacteria Rearrangement of *Clostridium* cluster IV (↓ *Faecalibacterium prausnitzii* and ↑ *Clostridium leptum*)
[58] Claesson et al. 2011	161 **elderly** Irish subjects (82F, 79M) (>65 years old) and a **control** group (5F, 4M) (28–46 years old)	Pyrosequencing with 454 system (16S rRNA V4 region)	↓ *Firmicutes proportion* ↑ *Clostridium* cluster IV (specially, *Faecalibacterium* spp.) ↓ *Clostridium* cluster XIVa	
[72] Wang et al. 2015	24 volunteers from China (14F, 10M) classified in **Group RC** (100–108 years old), **Group RE** (85–99 years old) and **Group CE** (80–92 years old)	Illumina MiSeq and qPCR (16S rRNA V4 region)	↑ α-diversity in centenarians ↑ *Escherichia, Roseburia* in centenarians ↓ *Lactobacillus, Butyricimonas, Coprococcus, Parabacteroides, Akkermansia, Sutterella, Faecalibacterium* in centenarians	
[76] Biagi et al. 2016	24 semi-supercentenarians (>105 years old, **group S**) (18F, 6M) vs. 15 young adults (22–48 years old, **group Y**) (8F, 7M) from Northern Italy. Results of **C and E groups** from Biagi et al. 2010 study were incorporated.	Illumina MiSeq and qPCR (16S rRNA V3-V4 region)	**Elderly**	**Centenarians**
			↓ *Bifidobacterium*	↓ *Bifidobacterium* in C ↑ *Bifidobacterium* in S
			↓ *Bacteroidaceae, Lachnospiraceae, Ruminococcacea* with age ↑ *Eggerthella, Bilophila, Akkermansia, Anaerotruncus, Christensenellaceae* and *Synergistaceae* with age	
[75] Kong et al. 2016	168 Chinese individuals (85F, 83M) grouped into **long-living group** (≥90 years old), **elderly group** (65–83 years old) and a **younger age group** (24–64 years old)	Illumina MiSeq (16S rRNA V3-V4 region)	↑ *Clostridium* cluster XIVa, *Ruminococcaceae, Akkermansia* and *Christensenellaceae* in long-living group ↑ microbial diversity in long-living group	
[64] Odamaki et al. 2016	367 Japanese volunteers between 0 and 104 years old (210F, 157M)	Illumina MiSeq and qPCR (16S rRNA V3-V4 region)	**Elderly**	**Centenarians**
			-	↑ microbial diversity in centenarians
			↑ *Bacteroidetes* (*Bacteroides* and *Clostridiaceae*) and *Proteobacteria* (*Betaproteobacteria* and *Deltaproteobacteria*) with age ↓ *Actinobacteria* with age ↑ *Porphyromonas, Treponema, Fusobacterium* and *Pseudoramibacter* with age	
[68] Bian et al. 2017	A total of 1095 healthy Chinese volunteers (533F, 562M) classified into eight groups according to their age (**children, adults, elderly, centenarians**)	Illumina MiSeq (16S rRNA V4 region)	**Elderly**	**Centenarians**
			↓ *Blautia* after 60 years old ↑ *Prevotella* and *Bacteroides* in 60–79 years old group	↓ *Bacteroides* and *Bifidobacterium* genera in the oldest groups vs. the youngest groups ↓ *Prevotella* and *Bacteroides* in centenarians
			↑ *Dorea, Clostridium insertae sedis, Clostridium sensu strictu 1, Marvinbryantia* and members of *Prevotella* in older subjects vs. young groups	
[66] Rahayu et al. 2019	80 Indonesian subjects (50F, 30M): **young group** (25–45 years old) and **elderly group** (≥70 years old)	Yakult intestinal flora-scan (YIF-SCAN) (qPCR method)	↓ microbiota concentration ↑ *Lactobacillus reuteri* and *Enterobacteriaceae* ↓ *Clostridium cocoides, Bacteroides fragilis, Clostridium leptum, Bifidobacterium, Prevotella* and *Lactobacillus plantarum*	
[73] Wang et al. 2019	187 elderly subjects from three groups of age (**65–70 years old**), (**90–99 years old**) and (**100+ years old**) from East China (120F, 67M)	Illumina MiSeq (16S rRNA V3, V4 and V5 regions)	**Elderly**	**Centenarians**
			↑ *Clostridium, Parabacteroides* and *Streptococcus* in 90–99 years old group vs. 65–70 years old group ↓ *Megamonas, Blautia* and *Coprococcus* in 90–99 years old group vs. 65–70 years old group ↑ *Bacteroides fragilis, Parabacteroides merdae, Ruminococcus gnavus, Coprococcus* and *Clostridium perfringens* in 90–99 years old group	↑ community richness (Ace and Chao1 index) in centenarians (90–99 years old and 100+ years old groups) ↑ *Ruminococaccaeae, Alistipes and Barnesiella* in 100+ years old group vs. 65–70 years old group ↓ *Lachnospira and Prevotella* in 100+ years old group vs. 65–70 years old group ↑ *Synergistetes, Verrucomicrobia* and *Proteobacteria* in longevity group vs. younger elderly group
[74] Kim et al. 2019	56 South Korea subjects classified in **centenarians** (95–108 years old) (27F, 3M), **elderly** (67–79 years old) (7F, 10M) and **adults** (26–43 years old) (3F, 6M)	Pyrosequencing with 454 system (16S rRNA V1–V3 regions)	**Elderly**	**Centenarians**
			↑ *Proteobacteria* in elderly vs. adults ↓ *Bacteroidetes* in elderly vs. adults	↑ *Verrucomicrobia* in centenarians vs. elderly ↑ *Proteobacteria, Actinobacteria* and *Verrucomicrobia* in centenarians vs. adults ↑ *Akkermansia, Clostridium, Collinsella, Escherichia, Streptococcus* and *Christensenellaceae* in centenarians vs. elderly and adults ↓ *Faecalibacterium* and *Prevotella* in centenarians vs elderly and adults

F: female; M: male. Changes (↑: increase; ↓: decrease) in the relative abundance of selected microbial taxa and in bacterial diversity with age. Names in bold denote each group for the corresponding study and are defined in the table.

**Table 2:** Dietary interventions, supplementation with probiotics and prebiotics and assessment of the effect of physical activity on elderly individuals, with or without the concurrence of metabolic diseases.

Reference	Subjects	Methodological Approach/Intervention	Main Findings in Gut Microbiota Composition	
**Dietary Interventions, Probiotics and Prebiotics**				
[82] Cancello et al. 2019	≥65 years old obese women (20)	Longitudinal study. Hypocaloric mediterranean diet (15 days, **DIET**), hypocaloric mediterranean diet + VSL#3 probiotic mix (15 days, **PRO**)	**DIET**	**PRO**
			↑ *Parabacteroides* ↑ *Bacteroides* ↑ *Christensenellaceae* *↑* *Methanobrebrevibacter* *↓* *Collinsela*	↑ *Akkermansia*
[83] Ghosh et al. 2020	65–80 years old (286 M and 326 F)	Randomized multicenter single-blind controlled trial. Mediterranean diet (**DIET**) and control group (**CON**) (1 year)	**DIET** ↑ *Faecalibacteriumn prausnitzii* ↑ *Roseburia* ↑ *Eubacterium* ↑ *Bacteroides thetaiotaomicron* *↓ Ruminococcus torques* *↓ Collinsela aerofaciens*	
[84] Mitchell et al. 2020	≥70 years old men (30)	Randomized single-blind controlled trial. Diet with the recommended protein intake (**RDA**) or twice the recommended protein intake (**2RDA**) (10 weeks)	No significant differences reported	
[85] Tran et al. 2019	Young healthy (13 M and 16 F). Community dwelling (>65 years old; 10 M and 18 F) and institutionalized elderly (>65 years old; 9 M and 13 F)	Randomized single-blind controlled trial. Prebiotic mix (**PRE**) vs. placebo (maltodextrin). Single daily dose over 26 weeks (Double dose for frail subjects).	**PRE** ↑ *Ruminoccocaceae* ↑ *Parabacteroides* ↑ *Clostridium* cluster IV ↑ *Phascolarctobacterium* (Significance reached for institutionalized subjects at 13 week)	
[86] Kim et al. 2020	≥65 years old (53)	Randomized double-blind, multicenter controlled trial. *Bifidobacterium bifidum* BGN4 and *Bifidobacterium longum* BORI (1 × 10^9^ CFU/dose) vs. placebo (two doses/day, 12 weeks)	**PRO** *↓ Clostridiales* *↓ Prevotellaceae* *↓ Allisonela* *↓ Eubacterium*	
**Physical activity**				
[87] Aoyagi et al. 2019	65–92 years old (140 M and 198 F)	Monitoring of daily physical activity by electronic device over 1 month. Comparative study between more active (>15 min/day at >3 METS;**MA**) and less active (<15 min min/day at >3 METS; **LA**)	**MA** ↑ *Bacillaceae* *↓ Fusobacteriaceae*	
[88] Castro-Mejía et al. 2020	>65 years old (109 M and 98 F)	Comparative study of patients stratified into high (**HF**) and low-physical fitness (**LF**)	**HF**	**LF**
			*Bifidobacterium adolescentis* *Christensenella*	Enterobacterales
[89] Taniguchi et al. 2018	62–76 years old men (33)	Randomised crossover trial. 3 sessions/week exercise in a cyclo-ergometer (**TRA**) and control sedentary period (**CON**; 5 weeks each)	**TRA** *↑ Oscillospira* (**CON** first only) *↓ C. difficile*	
[90] Morita et al. 2019	>65 years old women (32)	Non-randomized comparative trial. 12 weeks of 1 h/daily brisk walking (**TRA**) vs. 1 h/weekly session of trunk muscle training (**CON**)	**TRA**	**CON**
			*↑ Bacteroides* *↓ Clostridium subcluster XIV*	*↓ Clostridium subcluster IX*
[91] Yu et al. 2018	65–80 years old patients with hypertension (32 M and 24 F)	Comparative study of patients classified according to Weber’ system for functional capacity. Normal exercise capacity (**NE**) vs. reduced exercise capacity (**RE**)	**NE**	**RE**
			*↑ Betaproteobacteria* *↑ Ruminococcaceae* *↑ Faecalibacterium*	*↑ Escherichia_Sigella* *↑ Lactobacillales* *↑ Lachnospiraceae* *↑ Blautia*
[92] Zhu et al. 2020	Healthy elderly and overweight elderly (>61 years old) (897)	Comparative study. Overweight elderly daily exercise (**DROE**, 74) group vs. overweight elderly never or rare exercise group (**NROE**, 222)	**DROE** *↑ Turicibacteraceae* *↓ Pseudomonadaceae* *↓ Oxalobacteraceae* *↓* *Odoribacteraceae* *↓* *Barnesiellaceae*	

F: female; M: male. Changes (↑: increase; ↓: decrease) in the relative abundance of selected microbial taxa and in bacterial diversity with the interventions. Names in bold denote each group for the corresponding study and are defined in the table.
